# Post-Infectious Bronchiolitis Obliterans as a Model of Immune-Mediated Airway Fibrosis: A Pediatric Case Report

**DOI:** 10.3390/ijms27041804

**Published:** 2026-02-13

**Authors:** Rosamaria Terracciano, Martina Mazzoni, Alessandro Rossi, Fabio Antonelli, Pierluigi Vuilleumier, Daniela Melis, Annalisa Allegorico

**Affiliations:** 1 Department of Translational Medicine, Section of Pediatrics, University of Naples “Federico II”, 80129 Naples, Italy; rosamariaterracciano@libero.it; 2 Department of Medicine, Surgery and Dentistry “Scuola Medica Salernitana”, Pediatrics Section, University of Salerno, 84081 Baronissi, Italy; martmazz94@gmail.com (M.M.); dmelis@unisa.it (D.M.); 3 Unit of Pediatric Pneumology and UTSIR, Santobono-Pausilipon Children’s Hospital, 80129 Naples, Italy; a.rossi@santobonopausilipon.it (A.R.); fabantonelli65@gmail.com (F.A.); p.vuilleumier@santobonopausilipon.it (P.V.)

**Keywords:** post-infectious bronchiolitis obliterans, airway fibrosis, immune dysregulation

## Abstract

Post-infectious bronchiolitis obliterans (PIBO) is a rare but severe chronic lung disease of childhood, characterized by irreversible small-airway obstruction following severe lower respiratory tract infections early in life. The disease course is often progressive and associated with long-term respiratory morbidity, while effective disease-modifying therapies remain limited. We report the case of a young child who developed severe PIBO following adenovirus pneumonia complicated by prolonged respiratory failure and multisystem involvement. Diagnosis was based on persistent respiratory symptoms, characteristic radiologic findings, and poor response to conventional anti-inflammatory treatment. Given the severity of the clinical course and steroid-refractory disease, an individualized immunomodulatory strategy, including hydroxychloroquine, was initiated within a multidisciplinary framework. During follow-up, the patient showed progressive clinical improvement, with gradual weaning from continuous oxygen supplementation, fewer respiratory exacerbations, simplification of systemic therapies, and radiologic findings consistent with partial improvement. Although causal conclusions regarding treatment efficacy cannot be drawn from a single case, the overall disease trajectory appeared more favorable than typically reported in PIBO cohorts. This case supports the emerging view of PIBO as an immune-mediated airway fibrotic disorder and underscores the importance of integrating detailed clinical phenotyping with evolving molecular insights to inform future precision medicine in pediatric post-infectious airway disease.

## 1. Introduction

Post-infectious bronchiolitis obliterans (PIBO) is a severe and often irreversible chronic lung disease of childhood, typically arising as a long-term sequela of acute lower respiratory tract infections occurring early in life. The condition is characterized by persistent airway inflammation and progressive fibrotic obliteration of the small airways, resulting in fixed airflow limitation, ventilation heterogeneity, and significant long-term respiratory morbidity. Despite advances in supportive and symptomatic management, PIBO remains a challenging clinical entity due to its heterogeneous presentation, frequent diagnostic delays, and the absence of disease-modifying therapeutic strategies [1,2,3].

In addition to post-infectious etiologies, bronchiolitis obliterans may arise in a variety of non-infectious settings, including bronchiolitis obliterans syndrome following lung or hematopoietic stem cell transplantation, toxic inhalational injury, drug-related toxicity, and immune-mediated or systemic inflammatory conditions. Across these heterogeneous contexts, the disease converges on a shared pathological endpoint characterized by immune-driven epithelial injury, dysregulated repair, and progressive fibrotic remodeling of the small airways. Framing post-infectious bronchiolitis obliterans within this broader spectrum underscores its relevance as a paradigmatic model of immune-mediated airway fibrosis [2,4]. Although classified among rare pediatric chronic lung diseases, PIBO contributes disproportionately to long-term respiratory impairment, particularly when it develops during critical windows of lung growth and alveolarization. In this context, the immature lung appears particularly susceptible to maladaptive repair processes following severe infectious injury, leading to permanent structural remodeling rather than effective functional regeneration. This paradigm situates PIBO alongside other pediatric interstitial and small-airway disorders in which early-life insults exert lasting effects on lung architecture and function, emphasizing the importance of maintaining the balance between injury and repair during lung development [3].

From a pathobiological perspective, the transition from acute infectious injury to chronic airway obstruction in PIBO is increasingly recognized as an immune-driven process rather than a passive consequence of residual structural damage. Severe viral or bacterial infections can trigger a sustained inflammatory response in the distal airways, characterized by prolonged activation of innate immune cells and dysregulated adaptive immunity.

Persistent inflammation ultimately drives structural remodeling of the lower airways, resulting in small-airway fibrosis and fixed airflow obstruction, which may culminate in partial or complete obliteration of the terminal bronchioles. In pediatric patients, this process most commonly manifests as a constrictive pattern of bronchiolitis obliterans, characterized by concentric peribronchiolar fibrosis and poor responsiveness to corticosteroid therapy, reflecting the largely irreversible nature of the fibrotic remodeling [4].

Beyond structural airway changes, persistent immune activation profoundly interferes with normal epithelial repair mechanisms in the developing lung, shifting the balance from effective regeneration toward maladaptive fibrotic remodeling. The airway epithelium is not merely a passive target of inflammation but serves as an active regulator of tissue homeostasis, functioning as a critical interface between the host and the external environment. Viral-induced epithelial injury disrupts barrier integrity and engages pattern recognition and viral entry pathways, thereby amplifying cytokine release, neutrophil recruitment, and peribronchial inflammation [5,6].

Among adaptive immune pathways, the interleukin-17 (IL-17) axis has been implicated as a potential link between post-infectious inflammation and airway remodeling [7].

When inflammatory signaling persists, epithelial regenerative programs may become arrested in transitional or maladaptive states, resulting in impaired epithelial restitution and sustained profibrotic signaling. Recent molecular studies have demonstrated that transitional epithelial cell states and persistent injury programs play a pivotal role in driving fibrotic remodeling by sustaining immune–mesenchymal crosstalk and fibroblast activation [8,9]. Central to this process is the transforming growth factor-β (TGF-β) signaling axis, which integrates inflammatory cues with fibroblast differentiation into matrix-producing myofibroblasts, ultimately leading to excessive extracellular matrix deposition and irreversible airway narrowing [10].

Recent advances in high-resolution molecular profiling have further refined the understanding of fibrotic lung diseases by uncovering previously unrecognized cellular states and signaling networks involved in immune-mediated tissue remodeling. In particular, single-cell RNA sequencing and spatial transcriptomic approaches applied to idiopathic pulmonary fibrosis have revealed marked heterogeneity among epithelial cells, macrophages, and fibroblasts, identifying distinct profibrotic cell populations and transitional epithelial states that emerge in response to chronic inflammatory injury [11,12]. Despite these molecular insights, their application to pediatric post-infectious airway diseases remains limited, and PIBO continues to be primarily defined by clinical, functional, and radiologic criteria. This gap is particularly relevant given the unique susceptibility of the developing lung to immune-mediated injury and maladaptive repair. From a diagnostic perspective, characteristic chest computed tomography (CT) findings—including mosaic attenuation with air trapping, peribronchial thickening, bronchiectasis, and areas of ground-glass opacity—reflect heterogeneous small-airway involvement and represent a cornerstone of diagnosis [1]. Because histopathological confirmation is rarely feasible in pediatric patients, an integrated clinical–radiological approach, together with the exclusion of alternative causes of chronic small-airway disease, remains essential [3,4]. In this context, individual clinical observations can serve as valuable translational frameworks to integrate emerging molecular concepts with pediatric disease phenotypes.

Accordingly, we present a pediatric case of post-infectious bronchiolitis obliterans as a conceptual model to contextualize recent advances in immune-mediated airway fibrosis within a clinically relevant framework.

## 2. Case Description

We report the case of a female toddler (approximately 20 months of age) who developed severe post-infectious bronchiolitis obliterans following a life-threatening viral lower respiratory tract infection complicated by prolonged respiratory failure and multisystem involvement. The patient was born at term (39 + 5 weeks of gestation) by elective cesarean section, with a birth weight of 3050 g and an unremarkable perinatal course. Neonatal adaptation was normal, psychomotor development was age appropriate, immunizations were up to date, and family history was negative for chronic respiratory, autoimmune, thrombotic, or neuromuscular disorders. From approximately one year of age, she experienced recurrent episodes of wheezing. In December 2022, she had a documented SARS-CoV-2 infection with a mild clinical course.

At approximately 20 months of age, the patient presented to a local hospital with acute respiratory distress. Chest radiography showed focal pneumonia, and she was discharged on oral antibiotic therapy. However, within a few days, progressive respiratory deterioration prompted readmission and subsequent transfer to Santobono–Pausilipon Children’s Hospital for advanced care. Shortly after admission, she developed severe hypoxemic respiratory failure requiring transfer to the pediatric intensive care unit (PICU). Non-invasive ventilation was initially attempted, but due to worsening gas exchange and respiratory mechanics, endotracheal intubation and invasive mechanical ventilation became necessary. She required high-frequency oscillatory ventilation (HFOV) for approximately five days. Following extubation, delayed neurological awakening was observed. Brain magnetic resonance imaging (MRI) revealed extensive subacute ischemic–infarct lesions involving multiple vascular territories of the left posterior cerebral artery, affecting both cortical and deep structures, with evidence of thrombotic occlusion and mass effect. The radiological pattern was considered more consistent with a thromboembolic event than with post-infectious arteriopathy. Clinically, the patient developed right-sided hemiparesis. Antiplatelet therapy with acetylsalicylic acid was initiated, together with systemic corticosteroids, antiepileptic treatment with phenobarbital and levetiracetam, and supportive care. Given the history of ischemic stroke of unclear etiology, further diagnostic investigations were undertaken. Autoimmune testing, including antinuclear antibodies (ANA), extractable nuclear antigens (ENA), antineutrophil cytoplasmic antibodies (ANCA), anti-double-stranded DNA antibodies, and complement fractions C3 and C4, yielded results within normal limits. A broader immunorheumatologic work-up included a targeted genetic panel for immune system disorders, encompassing *ADA2*, which was negative. A comprehensive hematologic evaluation was performed; repeated assessments did not identify a definitive inherited thrombophilic disorder. Functional and genetic thrombophilia screening showed reduced protein S activity (40%) and mildly elevated factor VIII levels (167%) in the absence of pathogenic thrombophilic mutations. Antiplatelet therapy was continued at a dose of 4 mg/kg/day without complications. Immunological screening yielded results within normal limits. Microbiological investigations demonstrated persistent adenovirus positivity in respiratory specimens throughout hospitalization. Transient herpes simplex virus IgM positivity was observed, along with serological evidence of prior exposure to varicella zoster virus, rubella virus, and Epstein–Barr virus. Bronchoalveolar lavage cultures grew *Candida* species, and antifungal therapy with micafungin was initiated. Nutritional support initially required parenteral nutrition via central venous access.

Following clinical stabilization, on hospital day 12, the patient was transferred to an Intensive Rehabilitation and Functional Orthopedics Unit, where a structured neurorehabilitation program was initiated. In the context of global neurological improvement, multidisciplinary specialist reassessments supported the initiation of a gradual tapering of systemic corticosteroid therapy. In parallel, as oral feeding was progressively reintroduced, parenteral nutrition was gradually reduced and ultimately discontinued.

In the subsequent days, the patient developed worsening respiratory status, characterized by tachypnea, recurrent desaturation episodes—particularly during sleep—and bronchospasm. Repeated pulmonology consultations recommended continuous pulse oximetry monitoring and initiation of inhaled bronchodilator therapy. Given the limited clinical response, on hospital day 24, the patient was transferred to the Pediatric Pulmonology Unit for further diagnostic work-up. Upon admission, close clinical surveillance and strict monitoring of vital parameters were instituted. Owing to the presence of bilateral fine crackles on auscultation, predominantly at the left lung base, contrast-enhanced chest computed tomography angiography (CTA) was performed and demonstrated radiological worsening compared with a prior non-contrast chest CT obtained during the PICU stay. The examination revealed diffuse and marked peribronchovascular thickening extending to the peripheral lung fields bilaterally, associated with interlobular septal thickening and diffuse ground-glass opacities. These findings were more pronounced in the apical segment and middle lobe of the right lung and in the apical and lingular segments of the left lung. In addition, newly developed bilateral posterior bronchiectatic changes, absent on previous imaging, were identified. Overnight respiratory monitoring performed in room air revealed pathological nocturnal hypoxemia, with the hypoxemic index representing the most abnormal parameter, while apnea–hypopnea and mixed obstructive indices were only mildly altered. Given the persistent requirement for oxygen supplementation, nocturnal transcutaneous capnography and pulse oximetry were subsequently performed under minimal oxygen support. These assessments confirmed the absence of nocturnal hypercapnia while documenting a pathological oxygen desaturation index (ODI), with an ODI ≥ 3% of 7.6 events/hour at 0.2 L/min and an ODI ≥ 4% of 1.1 events/hour at 0.3 L/min, thereby confirming the indication for nocturnal oxygen therapy. As part of the extended respiratory diagnostic work-up, a next-generation sequencing (NGS) panel for interstitial lung diseases was performed. No pathogenic variants were identified in genes associated with pediatric interstitial lung disease. However, a heterozygous *MYBPC3* variant, c.3258G>A (p. Trp1086Ter), maternally inherited, was detected. This variant is classified as pathogenic according to American College of Medical Genetics and Genomics (ACMG) criteria and is associated with autosomal dominant hypertrophic cardiomyopathy with incomplete penetrance. No clinical or instrumental correlation was identified between this genetic finding and the patient’s respiratory phenotype or ischemic event, and current evidence does not support an association between *MYBPC3* variants and thromboembolic risk. In the context of the clinical course, persistent adenovirus detection, and characteristic radiologic findings, the overall picture was considered highly suggestive of post-infectious bronchiolitis obliterans. During hospitalization, the patient experienced an acute respiratory exacerbation characterized by bronchospasm and desaturation, requiring a brief readmission to intensive care (approximately 24 h), with reinitiation of intravenous corticosteroids and high-flow oxygen therapy. Respiratory viral testing detected rhinovirus, and parainfluenza virus type 3 was documented on nasopharyngeal aspirate in association with episodes of clinical and respiratory deterioration. Given the severity of respiratory disease, the poor response to corticosteroid boluses, and progressive radiologic abnormalities, an immunomodulatory strategy was considered. Following ophthalmologic evaluation and confirmation of normal baseline hepatic and renal function, hydroxychloroquine therapywas initiated, also with the aim of facilitating corticosteroid tapering in the setting of emerging steroid-related adverse effects, including systemic hypertension. Hydroxychloroquine treatment was initiated on 1 May 2023 at a dosage of 10 mg/kg/day, in the context of active disease and after multidisciplinary discussion. To date, the treatment continues. Given the progressive clinical improvement, the absolute daily dose was intentionally kept unchanged, without pro-kg adjustment despite physiological weight gain, resulting in a gradual reduction in the weight-adjusted dosage to approximately 6.9 mg/kg/day at the most recent follow-up. The monitoring strategy included regular clinical evaluations, periodic laboratory testing and ophthalmologic surveillance, with no significant adverse events reported. In light of the prolonged exposure to systemic corticosteroids, an endocrine evaluation of the hypothalamic–pituitary–adrenal axis was performed at the time of steroid discontinuation and demonstrated preserved function. Follow-up endocrine laboratory monitoring was planned. Nutritional status was closely monitored through a structured food diary and serial dietetic assessments, confirming adequacy of the nutritional regimen throughout the follow-up period.

From a cardiologic standpoint, echocardiographic evaluation demonstrated elevated pulmonary arterial pressures, with an estimated systolic pulmonary artery pressure of approximately 43 mmHg, in the setting of otherwise normal cardiac anatomy and preserved biventricular function. The increase in pulmonary pressures was interpreted as secondary to chronic hypoxic vasoconstriction and underlying parenchymal lung disease rather than primary cardiac or pulmonary vascular pathology. In this context, diuretic therapy with furosemide was initiated in association with optimization of respiratory support and oxygen therapy, given the coexistence of pulmonary hypertension, oxygen dependence, and severe pulmonary involvement. Pulmonary arterial hypertension-specific therapies were not initiated, as no structural or functional cardiac abnormalities were identified on echocardiography. Serial blood pressure monitoring revealed persistent systemic hypertension, considered iatrogenic and related to prolonged corticosteroid exposure. Accordingly, a blood pressure diary, dietary sodium restriction, and antihypertensive therapy with amlodipine were initiated at a dose of 0.05 mg/kg/day and subsequently increased to 0.1 mg/kg/day due to insufficient initial blood pressure control.

**Figure 1:**
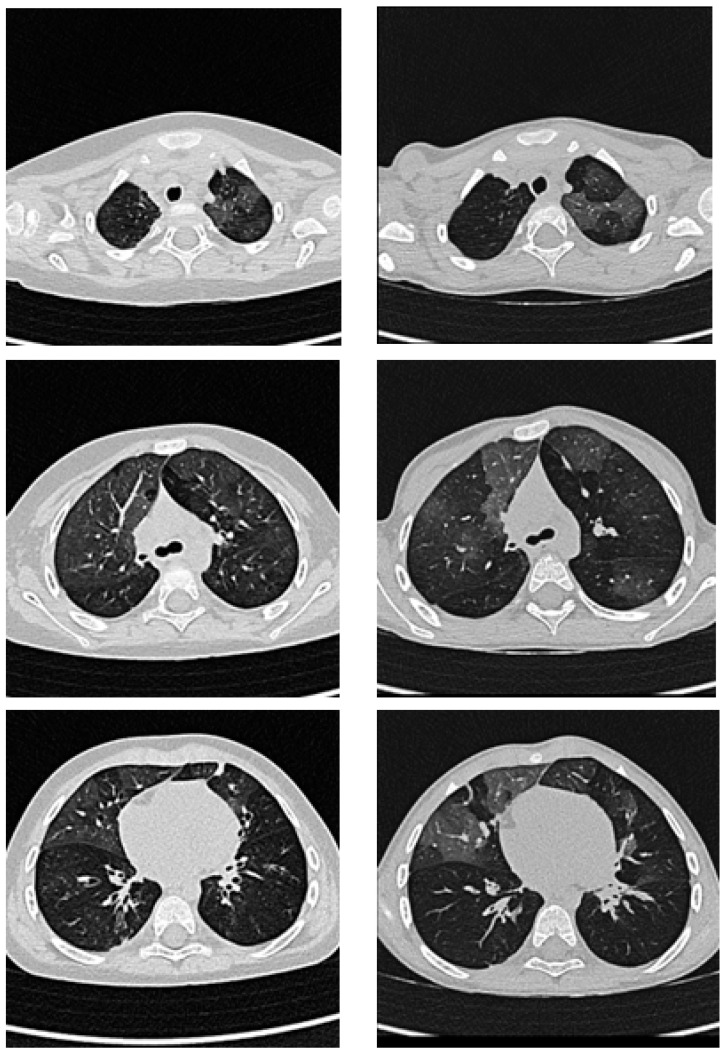
Improvement of lung involvement on contrast-enhanced chest computed tomography before and after treatment. (**Left**) panels: coronal chest CT images at the time of diagnosis. (**Right**) panels: corresponding images obtained at the same anatomical levels 24 months after treatment initiation, showing partial radiological improvement.

After a total hospital stay of 83 days, the patient was discharged in stable clinical condition with a comprehensive and structured therapeutic plan and a multidisciplinary follow-up program. At discharge, home therapy included inhaled fluticasone propionate, montelukast, hydroxychloroquine, azithromycin oral suspension administered three days per week, continuous low-flow oxygen therapy (24 h/day) and home pulse oximetry monitoring. Additional ongoing treatments included esomeprazole, acetylsalicylic acid, levetiracetam, phenobarbital, amlodipine, and furosemide, reflecting the multisystem involvement observed during the acute phase. Continuous oxygen supplementation was required for approximately six months after discharge, after which oxygen therapy was progressively reduced to nocturnal use only (approximately 0.2 L/min) and subsequently limited to use during respiratory exacerbations. The last documented use occurred during a respiratory exacerbation in September 2025 at a low flow rate (0.25 L/min). Serial pulse oximetry assessments documented a progressive improvement in nocturnal oxygen saturation in parallel with overall clinical recovery and respiratory stabilization. In the post-discharge period, recurrent episodes of bronchospasm prompted escalation of inhaled therapy from fluticasone monotherapy to combined fluticasone/salmeterol. Over time, respiratory exacerbations became less frequent and less severe. A follow-up chest CT performed approximately two years after the acute phase, prompted by the patient’s marked and sustained clinical improvement, demonstrated bilateral, symmetric, multifocal ground-glass opacities involving multiple lung segments, associated with peribronchial cuffing and residual basal disventilatory streaks with mild septal thickening. Although the overall distribution was comparable to earlier imaging, partial radiologic improvement was evident, consistent with the patient’s favorable clinical course (Figure 1). During follow-up, in parallel with progressive clinical improvement, resolution of oxygen dependence, and a reduction in pulmonary arterial pressures on serial echocardiographic assessments, furosemide was gradually tapered and ultimately discontinued in agreement with cardiology consultants, with final suspension in March Blood pressure values progressively normalized, allowing withdrawal of antihypertensive therapy with amlodipine in September. Antiplatelet therapy with acetylsalicylic acid, initially started after the ischemic event, was discontinued in February 2024 in agreement with hematology and neurology consultants, reflecting overall clinical stabilization and resolution of the initial indications for these treatments.

From a neurological perspective, structured follow-up continued after the ischemic event. Neurodevelopmental rehabilitation included neuropsychomotor therapy, speech therapy, and physiokinesitherapy, with gradual functional improvement over time. Periodic electroencephalographic monitoring initially demonstrated diffuse epileptiform abnormalities, which progressively improved. Antiseizure therapy was gradually de-escalated, with phenobarbital tapered to complete discontinuation in August 2023, while levetiracetam was continued with a planned stepwise dose reduction. A brain MRI performed in October 2025 confirmed chronic sequelae with gliomalacic evolution of the previously identified ischemic lesions, without evidence of new ischemic events. At the most recent follow-up, the patient was clinically stable on room air both during daytime and nighttime, requiring supplemental oxygen only sporadically during acute exacerbations. She demonstrated marked improvement in respiratory mechanics and chest auscultation findings, neurological function, ambulation, and speech, and continues maintenance therapy with hydroxychloroquine, inhaled fluticasone/salmeterol, levetiracetam, and azithromycin administered three days per week during the winter season, together with respiratory physiotherapy using a positive expiratory pressure mask, with further clinical improvement noted.

A concise timeline summarizing the major clinical events, therapeutic interventions, changes in respiratory support requirements, and key imaging timepoints is provided in Table 1.

## 3. Discussion

### 3.1. Clinical and Epidemiological Context of PIBO

PIBO represents a heterogeneous condition characterized by persistent small-airway inflammation and fibrotic remodeling following severe lower respiratory tract infection. In the present case, the combination of prolonged viral persistence, recurrent viral co-infections, and early radiologic progression toward bronchiectatic changes likely contributed to sustained airway injury and progressive airflow limitation.

Precise estimates of PIBO incidence and prevalence remain challenging due to heterogeneous diagnostic criteria, small cohort sizes, and geographic variability. Available data indicate a male predominance and an onset typically occurring before two years of age, during critical phases of lung development [1]. Epidemiologically, PIBO has been reported more frequently in the Southern Hemisphere, with increasing recognition in Northern Hemisphere countries, and shows higher prevalence in specific populations, suggesting that host-related genetic or epigenetic factors may contribute to disease susceptibility [1].

Clinically, PIBO is characterized by persistent respiratory symptoms that develop after an acute lower respiratory tract infection and fail to resolve over time. Affected children typically present with chronic cough, tachypnea, exertional dyspnea, wheezing, and recurrent respiratory exacerbations, often accompanied by hypoxemia and reduced exercise tolerance. These manifestations reflect fixed airflow obstruction and ventilation heterogeneity resulting from small-airway involvement and progressive fibrotic remodeling, contributing to long-term functional impairment and reduced quality of life [2].

### 3.2. Infectious Triggers and Disease Initiation

PIBO most commonly develops following severe lower respiratory tract infections occurring in early childhood. Adenovirus infection, as documented in our patient, is among the most strongly associated triggers, particularly serotypes 3, 7, 11, and 21, and is frequently linked to a more aggressive clinical course and poorer long-term outcomes [1].

Other implicated pathogens include Mycoplasma pneumoniae, respiratory syncytial virus, influenza and parainfluenza viruses, measles, and varicella, whereas rhinovirus has not been clearly linked to PIBO development. Sporadic cases have also been reported following severe SARS-CoV-2 infection, although its long-term contribution to PIBO pathogenesis remains incompletely defined [13].

Beyond the specific pathogen, the severity of the initial infectious insult appears to be a critical determinant of disease initiation. Prolonged hypoxemia, the need for respiratory support or mechanical ventilation, and an exaggerated inflammatory response during the acute phase have all been associated with an increased risk of subsequent airway injury and remodeling. These observations highlight the central role of host response severity in the pathogenesis of PIBO [1].

In this context, recent evidence has begun to clarify clinical and immunological risk factors predisposing children to the development of bronchiolitis obliterans following adenovirus pneumonia. In a large pediatric cohort, Yuan et al. identified prolonged wheezing duration, elevated serum lactate dehydrogenase (LDH) levels, and the need for respiratory support as independent predictors of bronchiolitis obliterans after adenovirus infection [14]. Prolonged wheezing may represent an early clinical manifestation of irreversible small-airway obstruction, whereas elevated LDH likely reflects the extent of acute lung injury and subsequent propensity toward aberrant repair. Similarly, the requirement for mechanical ventilation may act both as a marker of disease severity and as a contributor to ventilator-induced lung injury, further amplifying fibrotic remodeling processes.

Consistent with these observations, severe Mycoplasma pneumoniae has also been associated with an increased risk of subsequent bronchiolitis obliterans, particularly in younger children and in cases characterized by prolonged fever, hypoxemia, wheezing, elevated markers of tissue injury, and the need for intensive respiratory support. These findings further support the concept that the magnitude of epithelial injury and host inflammatory response, rather than the specific pathogen alone, represents a key determinant of PIBO development [15].

Immunological profiling in the same cohort revealed reduced proportions of circulating CD4^+^ and CD8^+^ T lymphocytes in children who developed bronchiolitis obliterans, suggesting impaired viral clearance and prolonged antigenic stimulation [1]. Persistent viral infection, particularly in the setting of co-infection with additional respiratory viruses, may therefore sustain inflammatory signaling and promote irreversible damage to the small airways. Together, these findings reinforce the concept that PIBO arises from a complex interplay among pathogen-related factors, host immune competence, and the magnitude of epithelial injury during the acute infectious phase.

### 3.3. Clinicopathological Correlates and Immune-Mediated Airway Remodeling

From a clinicopathological standpoint, PIBO comprises two principal histopathological patterns: a proliferative form, characterized by intraluminal granulation tissue and polypoid lesions, and a constrictive form, defined by concentric peribronchiolar fibrosis leading to progressive luminal narrowing or complete airway obliteration. In pediatric patients, constrictive bronchiolitis obliterans represents the predominant phenotype and is typically associated with poor responsiveness to corticosteroid therapy, reflecting the largely irreversible nature of fibrotic remodeling once established [4]. Although histopathological confirmation was not available in the present case, the integrated evaluation of clinical evolution, imaging findings, and limited response to systemic corticosteroids is consistent with this paradigm, supporting the concept that PIBO is driven by aberrant repair processes, culminating in fixed small-airway obstruction.

Beyond structural airway remodeling, persistent immune activation following infectious injury profoundly interferes with normal epithelial repair programs in the developing lung, shifting the balance from effective regeneration toward maladaptive fibrotic remodeling. The airway epithelium actively contributes to immune–repair dysregulation following viral injury: viral-induced epithelial damage disrupts barrier integrity and engages specific pattern recognition and viral entry receptors—including the coxsackievirus and adenovirus receptor (CAR), CD46, GD1a glycan, polysialic acid, and desmoglein-2—thereby amplifying cytokine release, neutrophil recruitment, and peribronchiolar inflammation [5,6]. Consistent with an immune-mediated disease model, children with PIBO exhibit sustained neutrophilic inflammation, with increased neutrophil counts in serum and bronchoalveolar lavage fluid, accompanied by elevated levels of pro-inflammatory and profibrotic mediators, including IL-1β, IL-6, IL-8, YKL-40, caspase-1, TGF-β, periostin, interferon-γ, β2-defensin, and the antimicrobial peptide cathelicidin (LL-37). These alterations occur alongside reduced expression of regulatory mediators such as IL-18, IL-27, KL-6, and microRNAs involved in epithelial homeostasis and immune modulation [1].

Among adaptive immune pathways, the interleukin-17 (IL-17) axis has emerged as a key mediator linking post-infectious inflammation to airway remodeling. IL-17-producing T cells promote sustained neutrophilic recruitment and amplify local cytokine networks, fostering a microenvironment that favors fibroblast activation and extracellular matrix deposition. Experimental studies have demonstrated that persistent IL-17 signaling contributes to chronic airway inflammation and fibrotic remodeling, whereas inhibition of this pathway attenuates both inflammatory burden and fibrosis, supporting a causal role for IL-17 in immune-mediated airway injury [7]. In parallel, biomarkers of tissue injury, such as lactate dehydrogenase (LDH), which correlate with disease severity during acute adenoviral infection, likely reflect the extent of epithelial damage and dysregulated immune activation, predisposing to maladaptive repair and subsequent PIBO development [13,16].

When inflammatory signaling persists, epithelial regenerative programs may become arrested in maladaptive transitional states, resulting in impaired epithelial restitution and sustained profibrotic signaling. Recent molecular studies have identified transitional epithelial cell populations, including Krt8^+^ progenitor-like states, as key drivers of immune–mesenchymal crosstalk and fibroblast activation through persistent injury programs [8,9]. Central to this process is the transforming growth factor-β (TGF-β) signaling axis, which integrates inflammatory cues with fibroblast differentiation into matrix-producing myofibroblasts, ultimately leading to excessive extracellular matrix deposition and irreversible airway narrowing [10]. Innate immune effector cells, particularly neutrophils and monocyte-derived macrophages, further contribute to sustained tissue injury through the release of proteases, reactive oxygen species, and profibrotic mediators, mechanisms well documented in experimental models of lung fibrosis and likely relevant to post-infectious airway remodeling in PIBO [17].

Taken together, these clinicopathological and emerging molecular observations support the conceptualization of PIBO as a paradigmatic model of immune-mediated small-airway fibrosis, rather than a static post-infectious sequela. Persistent immune dysregulation following severe early-life infection disrupts epithelial barrier integrity, impairs regenerative capacity, and promotes sustained immune–mesenchymal interactions, ultimately converging on irreversible fibrotic remodeling of the distal airways. Within this framework, PIBO shares key mechanistic features with other fibrotic lung diseases, including bronchiolitis obliterans syndrome following transplantation and idiopathic pulmonary fibrosis, while retaining pediatric-specific characteristics related to lung development and early immune responses [1].

### 3.4. Translational and Molecular Insights: Relevance to the Present Case

Although direct single-cell transcriptomic profiling of pediatric post-infectious bronchiolitis obliterans is currently lacking, emerging molecular studies provide valuable mechanistic frameworks for interpreting immune-mediated airway fibrosis. High-resolution single-cell RNA sequencing analyses in idiopathic pulmonary fibrosis have identified aberrant epithelial transitional states, profibrotic macrophage populations, and activated fibroblast subsets that collectively sustain chronic fibrogenesis through persistent immune–stromal crosstalk [11,12].

These findings support the concept that failure to resolve epithelial injury, rather than ongoing inflammation alone, represents a critical driver of irreversible airway remodeling.

Notably, initial attempts to apply single-cell approaches to bronchiolitis obliterans-related contexts are beginning to emerge. Recent single-cell transcriptomic analyses have demonstrated profound remodeling of epithelial cell states following injurious stimuli, characterized by activation of stress response pathways, impairment of differentiation programs, and induction of profibrotic signaling cascades [11,12].

Although these data are largely derived from models of fibrotic lung disease rather than PIBO, they suggest that airway and alveolar epithelial cells may adopt conserved maladaptive transcriptional programs across different forms of chronic bronchiolar injury [8].

Together, these observations support the hypothesis that PIBO shares fundamental molecular mechanisms with other fibrotic lung diseases, including immune-driven epithelial injury, arrested repair programs, and sustained fibroblast activation. Within this framework, the present pediatric case may be interpreted as a clinical correlate of these emerging molecular paradigms, highlighting the need for future studies applying single-cell and spatial transcriptomic approaches to pediatric PIBO to define disease-specific cellular drivers and to identify potential biomarkers or therapeutic targets.

### 3.5. Clinical Implications: Diagnosis, Management, and Long-Term Outcomes

Diagnosis of post-infectious bronchiolitis obliterans is primarily based on the integration of clinical history, persistent respiratory symptoms following a severe lower respiratory tract infection, functional evidence of fixed airflow obstruction, and characteristic radiologic findings, as histopathological confirmation is rarely feasible in pediatric patients [1]. Within this diagnostic framework, therapeutic management remains largely supportive and individualized, as no disease-modifying treatment has consistently demonstrated the ability to reverse established fibrotic airway remodeling. Current strategies aim to control inflammation, optimize respiratory support, prevent exacerbations, and monitor disease progression. Systemic corticosteroids are commonly used during the acute and subacute phases, while macrolides and inhaled therapies may be employed for their immunomodulatory and symptomatic effects [2,13]. However, evidence supporting a sustained impact on long-term outcomes remains limited. Consequently, alternative immunomodulatory strategies have been explored on a case-by-case basis, particularly in patients with severe or steroid-refractory disease. In our patient, the decision to introduce hydroxychloroquine was driven by the severity of the clinical course, the poor response to corticosteroid therapy, and its reported immunomodulatory and anti-inflammatory properties in pediatric inflammatory lung disorders.

Pulmonary hypertension represents a recognized complication of severe PIBO and is associated with increased morbidity. In our patient, elevated pulmonary arterial pressures were most likely secondary to chronic hypoxic vasoconstriction and underlying parenchymal lung disease, rather than primary vascular pathology. The subsequent improvement observed following optimization of respiratory support and diuretic therapy underscores the importance of early recognition and close cardiopulmonary monitoring in this patient population. The occurrence of an ischemic stroke during the acute phase of respiratory failure added further clinical complexity. Despite extensive hematologic, immunologic, and genetic investigations, no definitive prothrombotic condition was identified; however, severe systemic inflammation, hypoxemia, and critical illness may have contributed to a transient prothrombotic milieu. The incidental identification of a pathogenic *MYBPC3* variant raised additional genotype–phenotype considerations. Although *MYBPC3* mutations are classically associated with hypertrophic cardiomyopathy, there is currently no evidence supporting a direct association with PIBO or an increased thromboembolic risk. This finding underscores the growing likelihood of incidental findings with broad genetic testing and the need for careful multidisciplinary interpretation and follow-up.

From a functional standpoint, long-term follow-up studies indicate that many children with PIBO experience persistent respiratory impairment, prolonged oxygen dependence, and only partial recovery of lung function despite clinical stabilization, reflecting irreversible small-airway damage [18]. Against this backdrop, the clinical course observed in our patient is particularly noteworthy. A structured, prolonged, multidisciplinary follow-up was implemented, including nephrological, cardiological, neurological, and hematological evaluations. This approach allowed close monitoring of systemic involvement and supported a stepwise, individualized de-escalation of therapies in parallel with clinical improvement.

Following the introduction of hydroxychloroquine, the patient demonstrated a progressive and sustained improvement in respiratory status, enabling gradual weaning from continuous oxygen supplementation. Although she was initially discharged on 24 h oxygen therapy, the need for supplemental oxygen progressively decreased; the last documented use occurred during a respiratory exacerbation in September 2025, at low flow (0.25 L/min). Notably, oxygen supplementation was not required during two subsequent mild upper respiratory tract infections associated with common cold symptoms. Concurrently, respiratory and systemic pharmacological therapy was progressively simplified within the framework of structured multidisciplinary follow-up. Azithromycin oral suspension administered three days per week for approximately six months and subsequently discontinued, with reintroduction limited to the winter season as a preventive strategy. In parallel with clinical stabilization, a stepwise de-escalation of systemic therapies was achieved during follow-up, including withdrawal of antihypertensive, antiplatelet, antiepileptic, and diuretic treatments as their initial indications resolved. Since October 2025, the patient has also been enrolled in a respiratory physiotherapy program using a positive expiratory pressure (PEP) mask, with reported clinical benefit. Current maintenance therapy includes inhaled fluticasone propionate/salmeterol, hydroxychloroquine, and levetiracetam. As a consequence of physiological weight gain, the effective weight-adjusted dose progressively decreased, and at the most recent follow-up, after two years of therapy, the patient is receiving approximately 6.9 mg/kg/day. Follow-up chest computed tomography demonstrated bilateral but symmetric lung involvement, characterized by multifocal ground-glass opacities affecting multiple segments of both lungs, associated with peribronchial cuffing of segmental and subsegmental airways, predominantly in posterior and basal regions, and residual basal disventilatory streaks with mild interlobular septal thickening. Compared with previous imaging, these findings were interpreted as radiologic improvement, consistent with the patient’s favorable clinical trajectory. Although causal inferences regarding the role of hydroxychloroquine cannot be drawn from a single observation, the overall evolution appears more favorable than the prolonged oxygen dependence and fixed functional impairment commonly reported in PIBO cohorts. This case underscores the importance of structured, longitudinal, multidisciplinary follow-up, based on regular clinical and functional assessment, with serial pulmonary function testing when feasible and repeat chest CT reserved for clinically significant changes or extended intervals, thereby minimizing cumulative radiation exposure while ensuring adequate disease monitoring. The main clinical, epidemiological, radiological, and pathobiological features of post-infectious bronchiolitis obliterans are summarized in Table 2.

### 3.6. Limitations and Future Directions

This work has some limitations that should be acknowledged. First, the conclusions are derived from a single pediatric case, which inherently limits generalizability and precludes causal inference. In addition, direct molecular characterization of airway tissue was not feasible; mechanistic interpretations are therefore based on the integration of clinical observations with data from experimental models and adult fibrotic lung diseases. While this translational approach provides valuable insights, disease-specific molecular drivers of pediatric post-infectious bronchiolitis obliterans remain largely undefined.

Another important limitation is the lack of longitudinal molecular profiling, which restricts the ability to capture dynamic changes in immune activation, epithelial repair, and fibrotic progression over time. Furthermore, most high-resolution molecular insights currently available—such as single-cell and spatial transcriptomic data—originate from adult idiopathic pulmonary fibrosis or non-infectious models of airway injury, underscoring a critical gap in pediatric-specific data.

Future studies should prioritize the application of advanced molecular approaches, including single-cell and spatial transcriptomics, proteomics, and circulating biomarker profiling, to well-characterized pediatric PIBO cohorts. Integrating these technologies with detailed clinical phenotyping and long-term functional follow-up may enable the identification of disease-specific cellular drivers, prognostic biomarkers, and potential therapeutic targets. Ultimately, such efforts are essential to move beyond descriptive clinical frameworks toward precision medicine strategies for immune-mediated airway fibrosis in children.

## 4. Conclusions

PIBO represents a severe and largely irreversible consequence of early-life immune-mediated airway injury, in which persistent inflammation, impaired epithelial repair and fibrotic remodeling converge to cause permanent small-airway obstruction. By integrating clinical observations with emerging molecular paradigms, this case highlights PIBO as a translational model of immune-driven airway fibrosis in the developing lung. Advancing pediatric-specific molecular characterization of this condition will be essential to improve risk stratification, identify actionable biomarkers, and ultimately develop targeted therapeutic strategies aimed at modifying disease trajectory rather than merely managing its long-term sequelae.

## Figures and Tables

**Table 1:** Timeline of the clinical course, therapeutic interventions, respiratory support, and follow-up. This table summarizes the patient’s longitudinal clinical course, including major clinical events, modifications of medical therapy, changes in respiratory support, and the timing of key imaging and diagnostic investigations from the acute phase through long-term follow-up.

Timepoint	Clinical Status and Key Events	Respiratory Support	Treatments and Therapeutic Changes	Imaging and Investigations
**Birth–12 months**	Term birth; normal neonatal course and development	None	None	None
**~12–20 months**	Recurrent wheezing; mild SARS-CoV-2 infection (Dec 2022)	None	Intermittent bronchodilators	Chest X-ray during wheezing episodes
**~20 months (acute onset)**	Severe viral Lower respiratory tract infections with focal pneumonia	Progressive hypoxemia	Oral antibiotics	Chest X-ray: focal pneumonia
**Few days later**	Rapid deterioration; transfer to tertiary center; PICU admission	NIV → invasive mechanical ventilation → HFOV (~5 days)	Broad-spectrum antibiotics; systemic corticosteroids	Non-contrast chest CT during PICU stay
**PICU course**	Severe hypoxemic respiratory failure; delayed awakening; ischemic stroke	Invasive ventilation, then weaning	Phenobarbital; Levetiracetam; Acetylsalicylic acid (4 mg/kg/day)	Brain MRI: extensive left PCA ischemic–infarct lesions
**Early post-PICU**	Right-sided hemiparesis	Low-flow oxygen	Treatment unchanged	Hematologic evaluation, autoimmune and immunologic work-up
**Hospital day ~12**	Clinical stabilization; transfer to rehabilitation unit	Supplemental oxygen as needed	Gradual corticosteroid taper	None
**Hospital days ~18–23**	Worsening respiratory status; nocturnal desaturations and bronchospasm	Increasing oxygen need	Inhaled bronchodilators	Continuous pulse oximetry
**Hospital day ~24**	Transfer to Pediatric Pulmonology Unit	Persistent oxygen requirement	Treatment unchanged	Chest CTA: diffuse peribronchovascular thickening, ground-glass opacities, new bilateral bronchiectasis
**Pulmonology work-up**	Suspected PIBO; persistent adenovirus detection	Nocturnal oxygen required	Treatment unchanged	Overnight oximetry and transcutaneous capnography (pathological ODI, no hypercapnia) NGS panel for interstitial lung diseases
**Short ICU readmission**	Acute respiratory exacerbation (rhinovirus, parainfluenza 3)	HF oxygen (~24 h)	IV corticosteroid bolus	None
**1 May 2023**	Progressive disease despite steroids	Continuous low-flow oxygen	Hydroxychloroquine started (10 mg/kg/day)	Baseline ophthalmologic and laboratory monitoring
**Late hospitalization**	Pulmonary hypertension; steroid-related systemic hypertension	Continuous oxygen	Furosemide; amlodipine initiated	Echocardiography: sPAP ~43 mmHg
**Discharge (day 83)**	Clinical stabilization	Continuous oxygen (24 h/day)	Inhaled fluticasone propionate, montelukast, hydroxychloroquine, azithromycin (3 days/week), antiepileptics, aspirin	Cardiology, neurology, nephrology, and pulmonology follow-up
**First 6 months post-discharge**	Gradual clinical improvement	Oxygen progressively reduced to nocturnal only (~0.2 L/min)	No dose escalation of hydroxychloroquine despite weight gain	Serial nocturnal pulse oximetry
**September 2023**	Resolution of systemic hypertension	Nocturnal oxygen only	Amlodipine discontinued	None
**February 2024**	Stable neurological and hematologic status	Occasional nocturnal oxygen	Acetylsalicylic acid discontinued	None
**March 2025**	Improvement in pulmonary hypertension	Room air	Furosemide discontinued	Echocardiographic follow-up
**~2 years after acute event**	Marked and sustained respiratory improvement	Oxygen only during exacerbations	Maintenance therapy (HCQ, ICS/LABA)	Follow-up chest CT: partial radiologic improvement with residual ground-glass opacities
**Latest follow-up (2025)**	Stable respiratory and neurological condition	Room air	Ongoing maintenance therapy	Brain MRI: chronic ischemic sequelae, no new lesions

**Table 2:** Overview of the key epidemiological, clinical, radiological, and pathobiological characteristics of post-infectious bronchiolitis obliterans in children.

Domain	Characteristic	Description
Epidemiology	Incidence	Rare pediatric chronic lung disease; true incidence unknown due to underdiagnosis and heterogeneous criteria
	Age at onset	Typically <2 years of age, during critical phases of lung development
	Sex distribution	Male predominance reported in most cohorts
	Geographic distribution	More frequently reported in Southern Hemisphere; increasing recognition worldwide
Triggering event	Causative infection	Severe lower respiratory tract infection
	Most common pathogens	Adenovirus (especially serotypes 3, 7, 11, 21); also Mycoplasma pneumoniae, RSV, influenza, parainfluenza
	Severity of acute illness	Frequently associated with hypoxemia, PICU admission, and need for mechanical ventilation
Pathogenesis	Main mechanism	Persistent immune-mediated airway inflammation with maladaptive repair
	Histopathological pattern	Predominantly constrictive bronchiolitis obliterans (peribronchiolar fibrosis and airway obliteration)
	Immune features	Sustained neutrophilic inflammation; dysregulated innate and adaptive immunity
Clinical presentation	Onset	Persistent respiratory symptoms following apparent recovery from acute infection
	Main symptoms	Chronic cough, wheezing, tachypnea, exertional dyspnea
	Disease course	Chronic and often progressive; limited reversibility
	Oxygen requirement	Frequent prolonged oxygen dependence, especially during sleep or exacerbations
Functional findings	Pulmonary function tests	Fixed airflow obstruction; reduced FEV_1_; poor bronchodilator response (when testing is feasible)
Radiologic features	Chest CT findings	Mosaic attenuation, air trapping, peribronchial thickening, bronchiectasis, ground-glass opacities
	Distribution	Bilateral, often asymmetric; predominantly involving small airways
Diagnosis	Diagnostic approach	Clinical–radiological diagnosis; lung biopsy rarely feasible in children
	Key diagnostic criteria	History of severe infection, persistent symptoms, characteristic CT findings, exclusion of alternative diagnoses
Management	Therapeutic strategy	Largely supportive and individualized
	Pharmacologic options	Systemic/inhaled corticosteroids, macrolides, bronchodilators; immunomodulators in selected cases
	Disease-modifying therapy	None proven to reverse established fibrosis
Complications	Pulmonary hypertension	Secondary to chronic hypoxemia and parenchymal lung disease
	Long-term outcome	Persistent respiratory impairment common; variable clinical stabilization with structured follow-up

## Data Availability

The data supporting the findings of this case report are available from the corresponding author upon reasonable request, in accordance with ethical and privacy restrictions.

## Abbreviations

The following abbreviations are used in this manuscript:

PIBO	Post-infectious bronchiolitis obliterans
HFOV	High-frequency oscillatory ventilation
TGF-β	Transforming growth factor-β
PEP	Positive expiratory pressure
